# The Combination of Low-Frequency Ultrasound and Antibiotics Improves the Killing of In Vitro *Staphylococcus aureus* and *Pseudomonas aeruginosa* Biofilms

**DOI:** 10.3390/antibiotics11111494

**Published:** 2022-10-28

**Authors:** Lasse Kvich, Mads H. Christensen, Malgorzata K. Pierchala, Konstantin Astafiev, Rasmus Lou-Moeller, Thomas Bjarnsholt

**Affiliations:** 1 Costerton Biofilm Center, Department of Immunology and Microbiology, University of Copenhagen, DK-2200 Copenhagen, Denmark; 2 CTS Ferroperm Piezoceramics, DK-3490 Kvistgaard, Denmark; 3 Department of Clinical Microbiology, Rigshospitalet, DK-2100 Copenhagen, Denmark

**Keywords:** low-frequency ultrasound, flexible piezoelectric material, antibiotics, biofilm, *Staphyloccocus aureus*, *Pseudomonas aeruginosa*

## Abstract

Due to an increase in underlying predisposing factors, chronic wounds have become an increasing burden on healthcare systems worldwide. Chronic infections often contain biofilm-forming bacteria, which are challenging to eradicate due to increased antibiotic tolerance; thus, new and improved therapeutic strategies are warranted. One such strategy is the combination of ultrasound and antibiotics. Therefore, this study aimed to investigate the combinatory effects of low-frequency (50 kHz) ultrasound delivered by specially designed ultrasound patches using flexible piezoelectric material, PiezoPaint™, in combination with antibiotics against biofilms with *Staphylococcus aureus* and *Pseudomonas aeruginosa*. The reduction in viable cells in *S. aureus* and *P. aeruginosa* biofilms was evaluated post-treatment with fusidic acid, clindamycin, ciprofloxacin, and colistin in combination with ultrasound treatment. Two-hour ultrasound treatment significantly increased the bactericidal effect of all four antibiotics, resulting in a 96–98% and 90–93% reduction in *P. aeruginosa* and *S. aureus*, respectively. In addition, an additive effect was observed when extending treatment to 4 h, resulting in >99% and 95–97% reduction in *P. aeruginosa* and *S. aureus*, respectively. These results contrasted the lack of effect observed when treating filter-biofilms with antibiotics alone. The combined effect of ultrasound and antibiotic treatment resulted in a synergistic effect, reducing the viability of the clinically relevant pathogens *S. aureus* and *P. aeruginosa*. The modularity of the specially designed patches intended for topical treatment holds promising applications as a supplement in chronic wound therapy. Further studies are warranted with clinically isolated strains and other clinically relevant antibiotics before proceeding to studies where safety and applicability are investigated.

## 1. Introduction

Bacteria have two distinct lifeforms: single free-living cells, referred to as planktonic cells, and clustered aggregates, referred to as biofilms [1]. Traditionally, chronic infections are believed to contain biofilms, whereas acute infections are believed to harbor planktonic cells; however, recent evidence has shown that biofilms are present even in acute infections [2]. Infections harboring biofilms affect an increasing number of patients worldwide, and the total healthcare cost associated with biofilms is estimated to be 386.8 billion dollars [3]. Biofilms are estimated to be involved in up to 78.2% of chronic wounds [4], and due to increased antibiotic tolerance in biofilm infections and, thus, higher minimum inhibitory concentrations (MICs), chronic infections are challenging to treat with conventional clinical antibiotics [5,6]. An increase in lifestyle diseases such as diabetes is expected, contributing significantly to an increase in chronic wounds and creating a substantial future burden on healthcare systems [7,8]. Furthermore, chronic wound healing lasts 12 to 13 weeks and has a 70% recurrence rate, and failing to treat these wounds leads to loss of function or, worst case, amputation [9], emphasizing the patient-related costs. Collectively, these alarming statements highlight the need for novel and improved treatment strategies to improve the outcomes for patients suffering from biofilm infections such as chronic wounds. 

One such strategy is the combination of low-frequency ultrasound and antibiotics, which increases bacterial killing in biofilms [10,11,12]. As early as 1994, it was shown that low-frequency ultrasound (67 kHz) improved the penetration of antibiotics into biofilms [13], and a study on biofilm with the absence of antibiotics revealed that ultrasound (70 kHz) itself does not affect the viability of *Escherichia coli* and *P. aeruginosa* [14]. In addition to increasing the transportation rate of antibiotics in biofilms, it has been shown for *P. aeruginosa* that ultrasound increases the permeability of cell membranes, thus increasing the passage and effect of antibiotics [15]. However, despite previous findings of a synergistic effect between ultrasound and antibiotic treatment, there are currently no treatment options on the market that offer a wearable and flexible ultrasonic device for chronic wound treatment which integrates well with the soft and curved anatomy of the human body. This study used a piezoelectric material consisting of commercially available PZT (Lead Zirconate Titanate) ceramics in a polymer matrix (PiezoPaint™) to manufacture a patch that can emit ultrasound at frequencies and intensities that enable the above-described effect. Unlike traditional piezoceramic materials, which require processing temperatures of up to 1200 °C, PiezoPaint™ can be processed at less than 130 °C and can be printed using screen printing on flexible substrates such as wound-healing dressings, flexible PCB, polymeric substrates, and textiles, making it suitable for applications in the emerging field of epidermal electronics [16,17]. 

Some of the species that are most frequently isolated from chronic wound infections are *Staphylococcus aureus* and *Pseudomonas aeruginosa* [18]. Both species are commonly isolated from non-healing wounds, such as venous leg ulcers, with an estimated prevalence of 52% and 93% for *P. aeruginosa* and *S. aureus*, respectively [19]. Therefore, a PiezoPaint™-based flexible ultrasonic specially designed patch intended for topical treatment was manufactured to test the combined effect of low-frequency ultrasound (50 kHz) and antibiotics (fusidic acid, clindamycin, colistin, and ciprofloxacin) concerning the viability of *Pseudomonas aeruginosa* and *Staphylococcus aureus* when growing as biofilms. Overall, a synergistic time-dependent effect was observed between ultrasound and the antibiotics against *S. aureus* and *P. aeruginosa* filter-biofilms. This study is a proof-of-concept before proceeding to animal trials where safety and applicability are considered in relevant chronic wound animal models.

## 2. Materials and Methods

### 2.1. Strains and Growth Conditions 

This study includes in vitro testing with the laboratory strain *Pseudomonas aeruginosa (*ATCC 15692 from the Pseudomonas Genetic Stock Center) and *Staphylococcus aureus* (8325-4 [20]). Overnight (ON) cultures were prepared by propagating *P. aeruginosa* from the frozen stock onto lysogeny broth (LB) agar plates (2% agar; Substrate Department, University of Copenhagen, Copenhagen, Denmark) and incubating the plates at 37 °C. The following day, one colony was selected and grown in 5 mL LB at 37 °C, 180 rpm. *S. aureus* ON cultures were prepared similarly, but tryptic soy broth (TSB; BD, Franklin Lakes, NJ, USA) was used instead of LB.

### 2.2. Minimum Inhibitory Concentration (MIC)

The antibiotics investigated in this study were selected for their clinical relevance to the topical treatment of the strains studied [21,22,23,24]. The MIC of ciprofloxacin, fusidic acid, and clindamycin was determined with Etest^®^ strips (Biomérieux, Marcy-l’Étoile, France). ON grown colonies of both strains were suspended in 0.9% NaCl to a MacFarland 0.5 before spreading them onto Müeller–Hinton agar plates (2% agar; Substrate Department, University of Copenhagen, Copenhagen, Denmark). An Etest^®^ strip was placed on the bacterial lawn, and plates were incubated ON at 37 °C before determining antibiotic susceptibility. The MIC of colistin was determined with the broth microdilution method at the Clinic of Microbiology at Rigshospitalet, Copenhagen, Denmark. Both strains were sensitive to the selected antibiotics (colistin = 1.000 µg/mL, ciprofloxacin = 0.125 µg/mL, clindamycin = 0.094 µg/mL, fusidic acid = 0.190 µg/mL).

### 2.3. Filter-Biofilms 

In this study, filter-biofilms were used as a biofilm model. The method has previously been used to test the anti-biofilm properties of different wound dressings [25]. Filter-biofilms were prepared by spotting 10 µL of the ON culture to the center of a 0.2 μm, Ø25 mm Cellulose Nitrate Membrane Filter (Whatman^®^, Maidstone, UK). Subsequently, filter-biofilms were grown ON to create 24-hour-old biofilms and then transferred to the experimental plates containing 10× MIC of ciprofloxacin or colistin (Sigma-Aldrich, St. Louis, MO, USA) for *P. aeruginosa* and 10× MIC of fusidic acid and clindamycin (Sigma-Aldrich, St. Louis, MO, USA) for *S. aureus* filter-biofilms. Non-treated filter-biofilms were prepared similarly and transferred to experimental plates containing no antibiotics.

### 2.4. Ultrasound Patches

**Figure 1:**
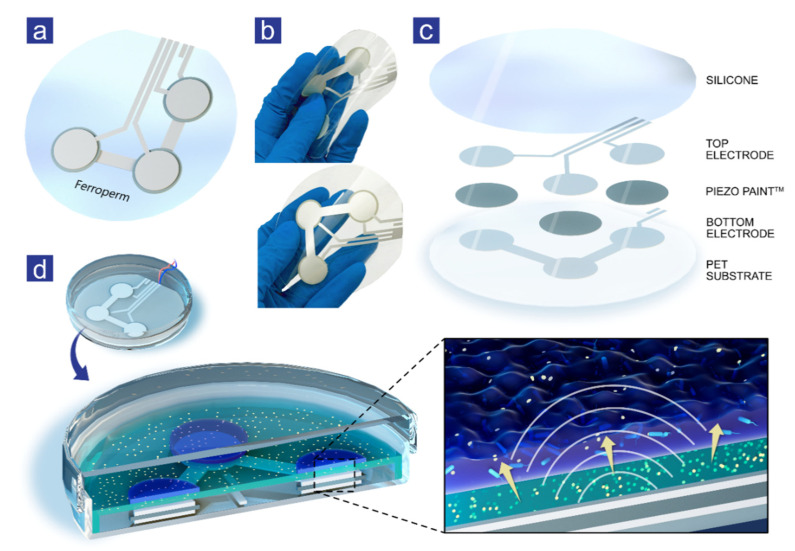
Ferroperm PiezoPaint™ patch. (**a**) Illustration presenting a three-element transducer screen-printed on a flexible PET substrate. (**b**) Photos of a flexible piezoelectric patch. (**c**) Expanded view exhibiting all layers of the ultrasound patch where the bottom silver electrode was screen-printed directly on a PET substrate, followed by deposition of PiezoPaint™ and silver top electrode. The sandwiched structure was protected with a silicone layer. (**d**) Image illustrating PiezoPaint™ patch in a Petri dish, covered with agar mixed with antibiotics on which 24-hour-old filter-biofilms were placed. The magnification represents the ultrasound waves generated by the PiezoPaint™ patch, which contribute to increased cell membranes' permeability and transport of antibiotics.

A multi-element transducer based on PiezoPaint™ (Ferroperm Piezoceramics, Kvistgaard, Denmark, patents for PiezoPaint™ can be accessed here [26,27]) was designed with a simple circular transducer with a sandwich-type structure. An active piezoelectric layer based on PiezoPaint™ of 17 mm diameter was screen-printed on 125 µm PET film using a commercial screen printer and sandwiched between the bottom and top silver electrodes of 19 mm in diameter (Figure 1a,b). The overall dimensions of the flexible transducer patch were 80 mm in diameter to fit a standard 90 mm Petri dish. The screen-printed electrodes and piezoelectric PiezoPaint™ layer were around 10 µm and 150 µm thick, respectively. The flexible multilayer transducer was poled following the CTS Ferroperm Piezoceramics procedure and approved for assembly, characterizing piezoelectric charge coefficient values *d*_33_ at the level of 40 pC/N. Wires were attached with conductive epoxy (EPO-TEK^®^ EJ2312) connecting the top and bottom electrodes, terminated with a standard BNC connector to connect to the driving system. The overall structure was assembled by casting medical-grade silicone (RTV118 FDA Silicone Adhesive) on top of the sandwiched structure as a protective silicone layer (Figure 1c).

### 2.5. Experimental Setup for Ultrasound Stimulation

The piezoelectric transducers were driven with a waveform generator (Agilent 33521A) applying a sinusoidal wave, amplified with an RF power amplifier (Trek PZD350A dual channel amplifier) with 100 times fixed amplification in voltage. The amplitude and frequency of the output signal were set on the waveform generator to 1.5 V and 50 kHz, respectively. The applied waveform was monitored through the monitor channel on the amplifier using an oscilloscope (Agilent DSO-X 3024A digital oscilloscope). The bacteria biofilms were exposed to acoustic waves for 2 and 4 h in combination with antibiotics. 

### 2.6. Experimental Setup with Ultrasound Patches

Ultrasound patches were placed in Petri dishes and covered with a 25 mL layer of LB broth with agar (2%) containing 10× MIC, and 24-hour-old filter-biofilms with *P. aeruginosa* and *S. aureus* were subsequently transferred to the plates right before the experiments were initiated. An illustration of the experimental setup is presented in Figure 1d. A frequency of 50 kHz was used, and 24-hour-old filter-biofilms were treated for 2 and 4 h in combination with antibiotics. In addition, both strains were treated for 4 h on agar plates with and without antibiotics, serving as controls. Outcomes were logarithmically transformed colony-forming units per milliliter (Log_10_ CFU/mL), and log-reduction was calculated between controls and ultrasound-treated filter-biofilms to determine the effect. 

### 2.7. Colony-Forming Units Per Milliliter (CFU/mL)

Determination of CFU/mL from filter-biofilms was performed on LB agar plates. Filters were transferred to 5 mL 0.9 % NaCl and vortexed for 60 seconds. Bacterial suspensions were then sonicated (5 min degas + 5 min sonication; Bransonic ultrasonic cleaner 2510, Emerson Electric, USA) before a ten-fold dilution series was performed in 0.9% NaCl. CFU was determined by plating three ten µL drops per dilution per biological replicate. 

### 2.8. Measurement of Temperature

The heat developed during 2- and 4-hour ultrasound treatments was measured in two ways: infrared measurements through the lids of the Petri dishes and thermocouples placed on the agar surface near the filter-biofilms. Infrared measurements were carried out with a Testo 805 thermometer (Testo SE & Co. KGaA, Titisee-Neustadt, Germany) every 30 min for *S. aureus* filter-biofilms during 2- and 4-hour treatments. Similarly, frequent measurements with the thermocouples (RS PRO Type K Thermocouple, Corby, UK) connected to the acquisition unit (Agilent 34972A LXI Data Acquisition/Switch Unit) were carried out over 4 h to measure the heat created near the filter-biofilms to evaluate temperature fluctuations. 

### 2.9. The combined Effect of Temperature and Antibiotics on the Viability 

Two antibiotics were tested in combination with temperature: fusidic acid for *S. aureus* and colistin for *P. aeruginosa*. Filter-biofilms and experimental plates with agar containing 10× MIC were prepared as described above. Both strains were treated for 4 h at different temperatures (37, 40, and 42 °C) in combination with antibiotics. The outcome was Log_10_ CFU/mL.

### 2.10. Statistics

The log-reduction was calculated for antibiotic-treated bacteria (controls) vs. the combined treatment of ultrasound and antibiotics. Data were tested for normality using the Shapiro–Wilk test before a one-way ANOVA was performed. Variation is presented as the standard deviation (SD), and a *p*-value < 0.05 was considered statistically significant. Tests and graphs were performed with Prism 6.1 (GraphPad Software, La Jolla, CA, USA).

## 3. Results

### 3.1. A synergistic Effect Was Observed between Ultrasound and Antibiotic Treatment

Four antibiotics were tested in combination with ultrasound: fusidic acid and clindamycin for *S. aureus* filter-biofilms and ciprofloxacin and colistin for *P. aeruginosa* filter-biofilms. An illustration of the ultrasound patches and the experimental setup is presented in Figure 1a,d. 

**Figure 2:**
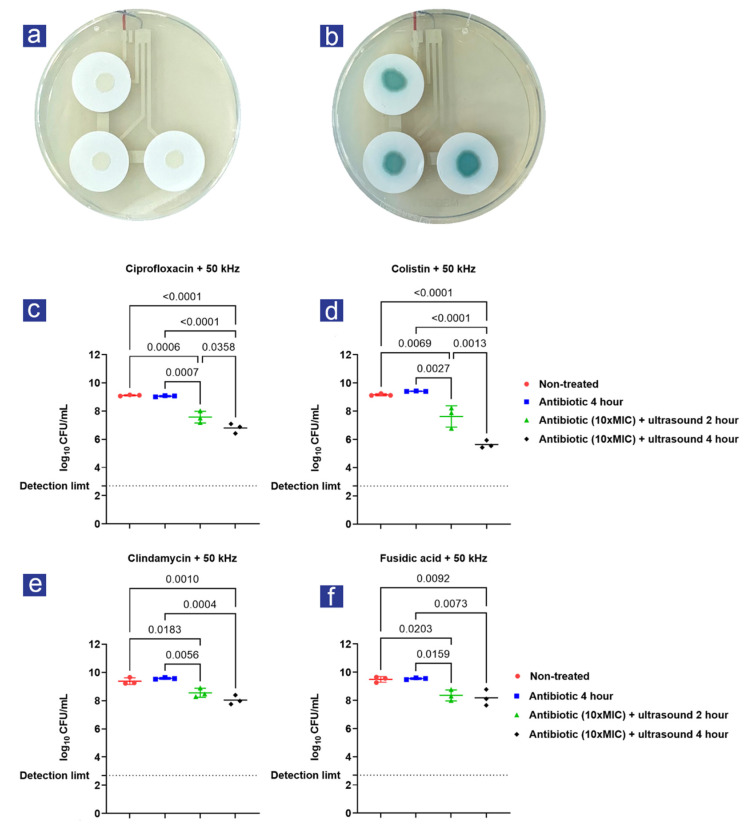
The combined effect of ultrasound and antibiotic treatment: (**a**,**b**) the final experimental setup with filter-biofilms of *S. aureus* (left) and *P. aeruginosa* (right) growing on the agar covering the ultrasound patches; (**c**,**d**) represent logarithmic-transformed colony-forming units per milliliter (Log10 CFU/mL) determined from *P. aeruginosa* filter-biofilms treated at 50 kHz for 2 and 4 h in combination with ciprofloxacin and colistin, filter-biofilms treated with and without antibiotics served as controls; (**e**,**f**) represent Log10 CFU/mL determined from *S. aureus* filter-biofilms treated at 50 kHz for 2 and 4 h with clindamycin and fusidic acid. Filter-biofilms treated with and without antibiotics served as controls. N = 3 for all experiments, and bars represent SD. Statistical tests were performed using one-way ANOVA; only significant differences (*p* < 0.05) are shown.

Filter-biofilms with *S. aureus* and *P. aeruginosa* were transferred to the experimental plates right before 2- and 4-hour treatments were initiated (Figure 2a,b). Antibiotic treatment with 10× MIC did not affect the viability of *S. aureus* and *P. aeruginosa* filter-biofilms (Figure 2c–f), as expected. However, a significant effect of both 2-hour and 4-hour treatment with 50 kHz ultrasound in combination with antibiotics was observed across all experiments compared to antibiotics alone (Figure 2c–f). In all cases, prolonged treatment (4 h) resulted in a significantly increased viability reduction compared to two-hour treatment; however, only for the colistin- and ciprofloxacin-treated filter-biofilms prolonged treatment with ultrasound resulted in a significant difference compared to two-hour treatment (Figure 2c,d). 

The combined effect of ultrasound and antibiotics compared to 4-hour antibiotic treatment is presented in Table 1. Antibiotic treatment compared to non-treated filter-biofilms is excluded, since a minor increase in Log_10_ CFU/mL was observed for colistin (0.25 ± 0.07 SD), fusidic acid (0.05 ± 0.19 SD), and clindamycin (0.19 ± 0.23 SD). Only ciprofloxacin treatment resulted in a log-reduction (0.06 ± 0.005 SD) compared to non-treated filter-biofilms. None of these differences were significant.

### 3.2. Heat Was Created during Ultrasound Treatment 

**Figure 3:**
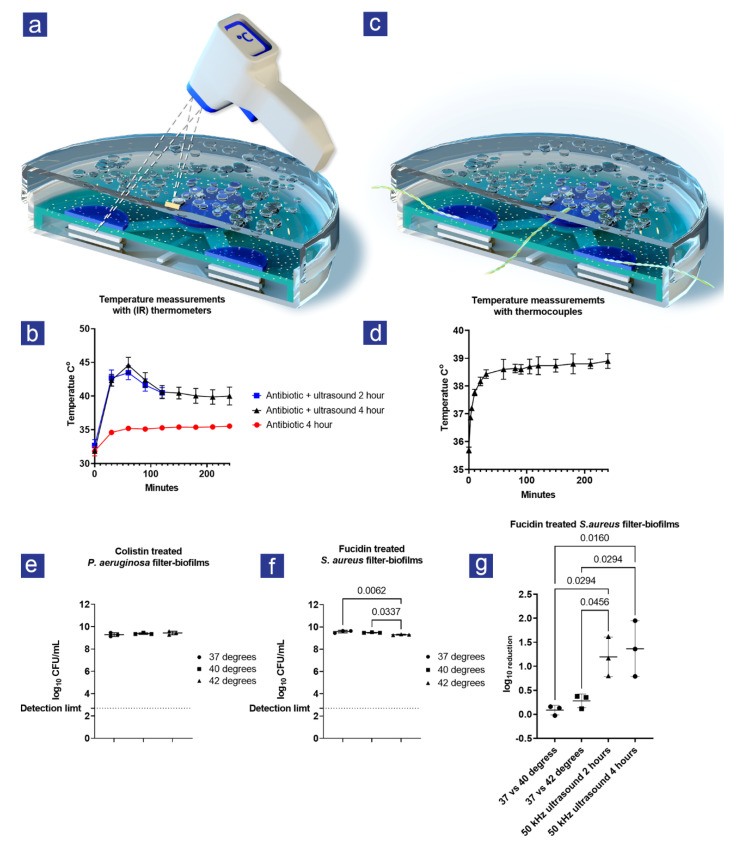
The effect of the temperature: (**a**) illustration of temperature measurement performed with an infrared (IR) thermometer; (**b**) temperature measurements over time measured with IR during 2- and 4-hour ultrasound treatment of *S. aureus* filter-biofilms; (**c**) illustrations of temperature measurement performed with type K thermocouples positioned at the surface level of the filter-biofilms; (**d**) temperature measurements over time with thermocouples at the surface level of the filter-biofilms during 4-hour ultrasound treatment; (**e**,**f**) graphs show logarithmic-transformed colony-forming units per milliliter (Log10 CFU/mL) for the combined effect of antibiotics and increase in temperature for *P. aeruginosa* and *S. aureus* treated filter-biofilms, respectively; (**g**) the logarithmic reduction (Log10 reduction) for temperature in combination with antibiotics and the combined effect of ultrasound and antibiotics. N = 3 for all experiments, except graph A (n = 6), and bars represent SD. Statistical tests were performed using one-way ANOVA; only significant differences (*p* < 0.05) are shown.

During initial testing, condensed water accumulated on the lids, possibly created by heat during the ultrasound treatment. Therefore, an infrared thermometer was used to measure the temperature every 30 min for *S. aureus* filter-biofilms (Figure 3a,b). When combined with ultrasound, the temperature peaked at 45 °C after one hour of treatment, after which it went down again to 40 °C. This temperature shift was not observed for the antibiotic-treated filter-biofilms without ultrasound treatment. Since the ultrasound energy levels are too low to generate heat, the assumption was that the heat came from the losses in the PiezoPaint™ patch. Because infrared temperature measurements provide a global measurement and the rise and fall of temperature contradict the behavior of a thermal system approaching equilibrium, they were considered unreliable. To further investigate the temperature increase, thermocouples were used to accurately measure the heat at the surface level of the filter-biofilms (Figure 3c,d). Similar to previous findings, an increase in temperature was observed over 4 h; however, the maximum temperature was 39 °C, and the temperature evolution resembles what is expected from a system moving toward thermal equilibrium. 

### 3.3. The Increase in Heat Could Not Explain the Combined Effect of Ultrasound and Antibiotics

A control experiment was conducted to assess if the increase in temperature had an additive effect on the viability of *S. aureus* and *P. aeruginosa* filter-biofilms using temperatures resembling what was observed at the level of the filter-biofilms. When the temperature increased, no effect was observed for colistin-treated filter-biofilms with *P. aeruginosa* (Figure 3e). However, a small but significant (*p* = 0.005) effect of temperature (37 °C vs. 42 °C and 39 °C vs. 42 °C) was observed for fusidic-acid-treated filter-biofilms with *S. aureus* (Figure 3f). The maximum difference (37 °C vs. 42 °C) between treatments was 0.28 log_10_ CFU/mL (95% CI: 0.12–0.44). Nevertheless, this difference was smaller than observed during the combined ultrasound and antibiotic treatment at 37 °C (Figure 3g), indicating that the rising temperature alone could not explain the observed effect. 

## 4. Discussion

Improved treatment strategies have become increasingly essential with the expected rise in chronic wounds and increased antibiotic tolerance and antimicrobial resistance in general [28,29]. In the present study, we investigated an alternative treatment for two of the most common species encountered in chronic wound infections: *S. aureus* and *P. aeruginosa.* In chronic wounds, *S. aureus* and *P. aeruginosa* are non-randomly distributed, with *S. aureus* positioned close to the wound surface, and *P. aeruginosa* situated more profoundly in the wound bed [28]. Understanding the spatial distribution of bacteria within wounds might help choose the proper treatment, such as topical treatment for superficial in situ located bacteria; however, the increased tolerance demands supplements to the conventional clinical treatment. Therefore, specially designed ultrasound patches intended for topical wound treatment were customized using PiezoPanit™ to fit a Petri dish to test the combined effect of ultrasound and clinically relevant topical antibiotics in this study. The overall goal of this study was to perform a proof-of-concept of the method. 

### 4.1. Main Findings

Previous studies have observed how ultrasound enhances the bactericidal effects of antibiotics [10,30,31]. The studies commonly show that ultrasound does not kill bacteria in itself but that the increased killing results from a synergistic effect between the antibiotic and the ultrasound. Similarly, we found that a frequency of 50 kHz increased the bactericidal effect of colistin and ciprofloxacin for *P. aeruginosa* filter-biofilms and fusidic acid and clindamycin for *S. aureus* filter-biofilms. The effect on viability was most pronounced in 2-hour treatments; however, prolonged treatment (4 h) increased the effect in all of the tests, indicating a time-dependent effect when treating filter-biofilms of *S. aureus* and *P. aeruginosa*. 

The combined effect of ultrasound and antibiotic treatment was observed for all four antibiotics, even though they belong to four different antibiotic classes. A higher effect was observed for *P. aeruginosa*, and a plausible explanation is that the antibiotics used were bactericidal, whereas the antibiotics used for *S. aureus* can possess both bacteriostatic and bactericidal actions [32,33]. Common for many antibiotics is that they require a certain amount of metabolic activity for the antibiotic to be efficient [34]. In combination with antibiotic impermeability through the matrix and upregulation of, e.g., efflux pumps, the dormancy of cells embedded in the biofilm is one of the reasons why biofilm-related infections are so difficult to eradicate using conventional antibiotics [35]. Altering the metabolic state of bacteria in biofilms to increase the efficacy of an antibiotic is an exciting approach. Carmen et al. showed how ultrasound (70 kHz) increased gentamicin transport through *P. aeruginosa* and *E. coli* biofilms [36]. It is plausible to assume that ultrasound also increases the oxygen diffusion and transport of nutrients, thus altering the metabolic activity of the bacteria. In addition to increased transport through the biofilms, another dominant theory is that increased permeability of the bacterial cell membranes due to ultrasound treatment allows increased passage of antibiotics [37]. Though speculative, these mechanisms may apply to the experimental setup in this study and should be investigated in future experiments to understand the mechanisms better. 

Lastly, an increase in temperature was observed (37 to 39 °C) at the level of the filter-biofilms, and a combination of increased temperature and antibiotic treatment demonstrated increased killing for *S. aureus* filter-biofilms treated with fusidic acid. Nevertheless, it is not our impression that this difference can explain the bacterial killing observed when filter-biofilms were treated with ultrasound in combination with the same antibiotics. However, an additive effect cannot be ruled out when treating *S. aureus* filter-biofilms with fusidic acid. 

### 4.2. Limitations and Strengths

The filter-biofilm setup lacks several in vivo characteristics, and this model does not grasp wound-healing dynamics, which is a well-known problem for several biofilm models [38]. Nevertheless, findings from this study strengthen the conclusion that ultrasound in combination with clinically relevant antibiotics results in synergistic cooperation, resulting in the enhanced killing of two bacterial species known to dominate in chronic wounds. In addition, the observed effect in this study occurred through a layer of agar, suggesting the plausible applicability of this method in a clinical setting where *S. aureus* and *P. aeruginosa* are situated in different depths of the wound [28]. 

### 4.3. Future Perspectives

As many as 50% of hospital patients have wounds, accounting for around 3% of costs in the healthcare system [39]. It is relatively inexpensive to manage simple surgical incisions; however, if an infection occurs, the cost rises substantially. It is estimated that the necessity of treating a surgical site infection adds around 11 days of in-patient hospital stay [40]. Hospitalization accounts for around 50% of the total cost, while nurse time and dressings contribute to around 35% and 15% of overall costs, respectively [39]. Therefore, it is essential to focus on the main expense drivers and efficiently manage the patient by introducing an effective intervention. In addition, beneath all statistics and figures showing the impact on the healthcare system, one should remember the individual patients affected by wounds. Many patients have elevated levels of psychological distress correlated to wounds manifested in extended hospital stays, including pain, social isolation, and anxiety [41], adding to the necessity of improved treatment options for chronic wounds.

**Figure 4:**
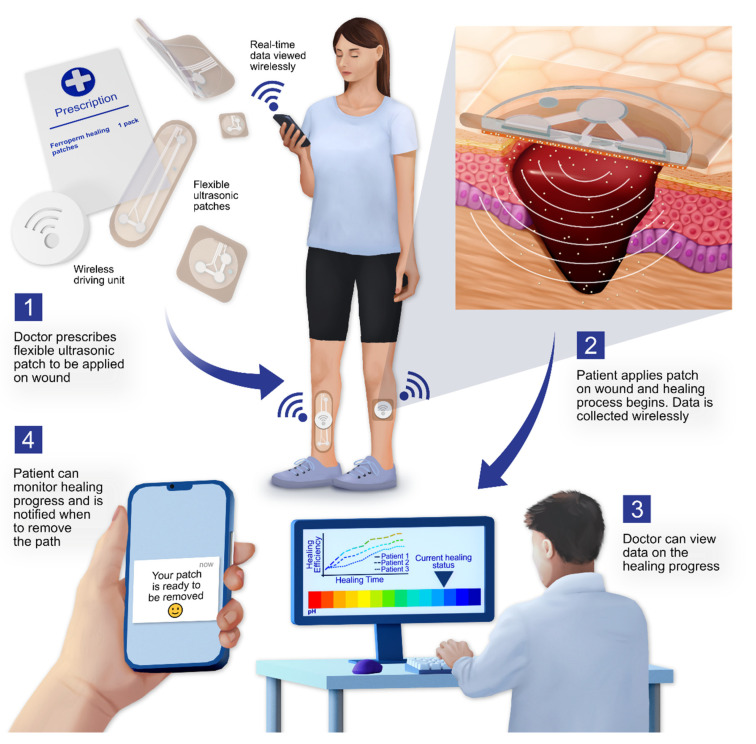
Treating wounds is a significant part of the healthcare financial plan with a high contribution from the cost of hospitalization and nurse time. Indeed, the focus should be on reducing these expense drivers by managing patients more effectively. A combination of passive dressings with flexible piezoelectric patches can address such shortcomings where patients can apply patches themselves at home. Clinicians and patients can monitor the treatment thanks to integrated tracking sensors.

As demonstrated in this study, the flexible ultrasonic patch could potentially address such shortcomings to give rise to a wound-healing therapy that can combine passive dressings with active therapy by introducing ultrasound based on the flexible PiezoPaint™ technology. Healthcare professionals could prescribe ultrasonic patches as a “bedside” therapy where patients apply the patches themselves directly at home. Both the clinician and the patient can monitor the progress of the treatment thanks to the wireless communication system allowing to acquire the data from integrated tracking sensors capable of detecting important biomarkers such as pH (Figure 4). A derived effect of bedside therapy is that hospital beds can be offered to other patients while clinicians and nurses could allocate their resources to treat additional patients. 

Ultrasound treatment used in this study showed a significant decrease in bacterial viability after 2 h and a further decrease at the 4-hour mark, potentially contributing to an increase in the healing process of chronic wounds, as described in comparable ultrasonic techniques [42,43,44]. Notably, previous techniques do not offer similar fidelity as the adaptable PiezoPaint™ patch that can conform well to the curved anatomy of a human body. In addition, it is noteworthy that most in vivo studies involve continuous treatment for 24–48 h, whereas we foresee a shorter treatment time using the adaptable PiezoPaint™ patch.

Nevertheless, findings from this study need to be further tested and validated in relevant chronic wound animal models. For instance, other clinically relevant antibiotics such as gentamicin, polymyxin b, mupirocin, and vancomycin should be tested to determine if ultrasound likewise enhances the effect of these antibiotics, as observed for the antibiotics used in this study. In addition, although using two highly relevant bacterial species for chronic wounds, future validation of these results should include testing with clinical isolates from chronic wounds. Based on the results from those experiments, we hope to proceed to clinical trials in the future to validate findings and investigate the safety and applicability of this method as an improved and efficient wound therapy. 

## 5. Conclusions

Continuous, low-frequency ultrasound (50 kHz) in combination with ciprofloxacin, colistin, fusidic acid, and clindamycin effectively enhances the bacterial killing of *P. aeruginosa* and *S. aureus* biofilms. The exact influence of ultrasound therapy on the efficacy of antibiotics is yet to be determined and requires further research. Future research should also include testing with other clinically relevant antibiotics and clinical isolates from chronic wound infections. Nevertheless, this proof-of-concept study shows that the combination of ultrasound and antibiotics has the potential to counteract some of the problems encountered with chronic infections, specifically for chronic wounds where the applicability of ultrasound through flexible, wearable patches seems plausible. 

## Figures and Tables

**Table 1:** The combined effect of ultrasound and antibiotic treatment compared to 4-hour antibiotic treatment.

	Log-Reduction	% Reduction	*p*-Value
**Ultrasound + colistin 2-hour**	1.78 ± 0.63	98.34%	0.003
**Ultrasound + colistin 4-hour**	3.77 ± 0.22	99.98%	<0.001
**Ultrasound + ciprofloxacin 2-hour**	1.49 ± 0.37	96.76%	0.001
**Ultrasound + ciprofloxacin 4-hour**	2.26 ± 0.25	99.45%	<0.001
**Ultrasound + fusidic acid 2-hour**	1.19 ± 0.33	93.54%	0.016
**Ultrasound + fusidic acid 4-hour**	1.37 ± 0.47	95.73%	0.007
**Ultrasound + clindamycin 2-hour**	1.02 ± 0.23	90.45%	0.006
**Ultrasound + clindamycin 4-hour**	1.54 ± 0.22	97.12%	<0.001

## Data Availability

The data generated in this study are available from the corresponding author.
